# Association between Burnout, Job Dissatisfaction and Intention to Leave among Medical Researchers in a Research Organisation in Malaysia during the COVID-19 Pandemic

**DOI:** 10.3390/ijerph191610017

**Published:** 2022-08-14

**Authors:** Halizah Mat Rifin, Mahmoud Danaee

**Affiliations:** 1 Institute for Public Health, National Institutes of Health, Ministry of Health Malaysia, Shah Alam 40170, Malaysia; 2 Department of Social and Preventive Medicine, Faculty of Medicine, University Malaya, Kuala Lumpur 50603, Malaysia; mdanaee@um.edu.my

**Keywords:** intention to leave, burnout, job satisfaction, researchers

## Abstract

Employee turnover could affect the organisation’s performance. Job dissatisfaction and burnout have been identified as factors influencing the intention to leave. Thus, this study aimed to determine the level of intent to leave, and predictors associated with intention to leave among medical researchers in Malaysia. A cross-sectional, stratified random sampling study was conducted among researchers in a research organisation under the Ministry of Health. Respondents answered an online questionnaire that included sociodemographic information, job dissatisfaction, burnout, and intention to leave. A total of 133 researchers participated. More than one-third (41.4%) of the researchers had a moderate and high level of intention to leave. Burnout and job dissatisfaction were identified as significant predictors. Burnout was noted to have a positive relationship with the intent to leave (β = 0.289, 95% CI (B): 0.287, 1.096). Meanwhile, job satisfaction was found to have a negative relationship with the intention to leave (β = −0.348, 95% CI (B): −0.768, −0.273). Burnout among researchers is quite worrisome as more than two-thirds of the researchers experienced moderate to high burnout. Reducing burnout and job dissatisfaction would increase work performance and produce high-quality research output, hence decreasing the turnover rate.

## 1. Introduction

Employee turnover rates could significantly impact the organization [1]. In the healthcare sector, for example, the quality of local healthcare services is influenced by the turnover of the employees [2]. Inadequate health workers lead to skills imbalance among workers, poor motivation, and poor performance of health workers especially in low and middle-income countries [3,4]. This could affect the delivery and extension of health programmes. Moreover, the selection, recruiting, training, and orientation of new employees are also costly [5].

Turnover is different from intention to leave, as turnover is an actual act of the individuals leaving the organisation, meanwhile, the intention to leave is the individual’s perception to quit the organisation. A direct turnover predictor is the intention to leave [6] and considered to be the strongest predictor among healthcare workers [7].

The reasons for the intention to leave identified include burnout, commitment to an organisation, job satisfaction, organisational factors, working factors, employee factors, external factors, and human resource management practices [8].

Globally, there is an estimated shortage of 7.2 million health professionals, and it is predicted to double in the next few decades [9]. About 36–40% (a sizable proportion) of healthcare professionals in Malaysia intended to leave their organisation before reaching pensionable age [10]. Among the reasons identified were interpersonal relationships at work, economic reasons, and “personal factors”.

Researchers are part of the professional staff. The professionals, including the medical researchers of all specialties, are prone to mental illness, which includes burnout and distress, due to their work [11,12]. This may be due to researchers often having to multi-task (budgeting for a research project, grant application, manage personnel, and handle other responsibilities with time constrains, thus giving them limited time to perform research-oriented tasks and ponder big ideas [11]). Burnout and distress symptoms among medical researchers were also linked to scientific disintegrity and publication pressure. The prevalence of stress was 77.4%, and the risk of significant distress was 5.1% among medical researchers in Italy [11]. Researchers’ mental health issues may lead to their desire to leave the organisation. The world was still dealing with a COVID-19 pandemic at the time of this study, and Malaysia was placed under Movement Control Order (MCO). The COVID-19 pandemic continues to significantly increase demand among healthcare workers, who are already overwhelmed with work environment and other responsibilities [13]. Many restrictions were imposed at the time, which disrupts the usual pre-pandemic research norms [14].

Public health specialists, medical officers, pharmacists, research officers, microbiologists, nutritionists, dieticians, and science officers are among the different medical professions working as researchers in the research organisation in this study, which is also governed by the Ministry of Health (MOH). As the research organisation’s mission is to conduct effective and high-impact health-related research, this study is among the first to look at the association between job satisfaction, occupational burnout, and intention to leave among researchers in Malaysia and its identification of their demographics.

Thus, the general objective of this study is to determine the level of intention to leave and predictors of intention to leave among researchers during COVID-19 pandemic. The specific objectives include: (i) to evaluate the level of intention to leave among researchers; (ii) to investigate the relationship between sociodemographic factors and intention to leave among researchers; and (iii) to determine the effects of burnout and satisfaction of job on intention to leave among researchers.

The findings of this study could help decision-makers to develop strategies that prevent employee turnover and so reduce costs in the long run. In addition, it could lead to more productivity and an increase in performance in terms of producing better quality research output.

## 2. Materials and Methods

A cross-sectional study was conducted with random sampling based on a list of the current researchers working in a research organisation under the Ministry of Health.

### 2.1. Participants

The subjects were recruited among the medical researchers who fulfilled the inclusion criteria from May 2021 until July 2021. This study was conducted in a research organisation under the Ministry of Health.

The inclusion criteria include (i) permanent technical staff doing research-oriented tasks grade 41 and above who worked at least 6 months in the research organisation.

The exclusion criteria include (i) technical staff who are doing administrative work only and (ii) technical staff on leave (long medical leave, maternity leave, sabbatical leave, study leave or attending courses during the data collection period of 3 months).

The sample size calculation for this study was done using G* Power Software [15]. The level of significance was set at (α) 0.05, and the power of study (1-β) was set at 80%. The sample size estimation was based on the number of predictors, which are the variables associated with the intention to leave the organisation. Ten variables were considered for the estimation of the sample size. These include age, gender, marital status, ethnicity, educational level, research working experience, household income, job satisfaction, burnout, and intention to leave. The sample population was 149 based on the sample size recommendation from G* Power Software (118 + 25% non-response rate) out of 588 researchers in the organisation. An additional 25% of the sample size was added to secure the final sample size in case of refusal.

Stratified random sampling was applied in this study. The list of names of researchers working in each division was obtained from the human resource management department. The proportionate stratification was done among eight divisions. The division with the higher proportion of researchers had a higher number of researchers selected. The selection of researchers within the strata was made using simple random sampling. It used computer-generated simple random sampling [16]. After the selection was made, the list of respondents was obtained. Overall, there were about 588 researchers working in the research organisation. Of those, 149 researchers were selected after considering 25% of the non-response rate.

Ethical approval for this study was obtained from the Medical Research and Ethics Committee (MREC) from the National Institutes of Health, Ministry of Health Malaysia [NMRR-20-2350-56886 (IIR)]. The study’s objectives were informed to the respondents, and consent was obtained prior to participation. The participant’s information gathered from this study were kept confidential.

### 2.2. Instrumentation

An online questionnaire using Google Form was created to collect responses anonymously. The respondents were emailed with the invitation links of the survey questionnaires. The consent and information sheet were also emailed together. A reminder on the duration of data collection was sent out periodically. To optimise the response rate, other methods were also applied. The methods included the distribution of QR codes of the survey questionnaire and personal invitation through WhatsApp messages.

The questionnaires were divided into four sections.

#### 2.2.1. Sociodemographics

The first section covered sociodemographic questions such as gender, age, marital status, ethnicity, educational level, research working experience (in years), and household income.

#### 2.2.2. Job Satisfaction

The short form Minnesota Satisfaction Questionnaire(MSQ) was adopted to investigate job satisfaction [17]. It consists of 20 items from the long-form MSQ that best represent each of the 20 scales. The reliability of the short form MSQ was good (Cronbach alpha = 0.85–0.91) in the previous study [18] and 0.86 during this current study. A five-point Likert scale ranging from (1—Very dissatisfied, 2—Dissatisfied, 3—Neutral, 4—Satisfied, to 5—Very satisfied) was used to measure job satisfaction. The score for job satisfaction was categorised into three categories: high (76–100), moderate (26–75), and low (0–25) [19]. A score of 26–100 (moderate and high degree satisfaction of job) indicates that the individual was satisfied with her/his job. In contrast, 25 and below indicates that the individual is dissatisfied with their job [19].

#### 2.2.3. Burnout

The Oldenburg Burnout Inventory (OLBI) questionnaire was adopted to assess burnout [20]. It has an affective component of exhaustion, and cognitive and physical dimensions. OLBI has positive and negative words. It is suitable for employees of any job. The Cronbach’s alpha values was 0.63 in the previous study [20] and 0.76 during this current study.

OLBI consists of 16 positive and negative formulated items that evaluate the two dimensions of burnout. Items are scored on a four-point scale from strongly agree (1) to strongly disagree (4). The cut-off point was indicated by a score of ≥2.25 for high exhaustion and a score of ≥2.10 for high disengagement [21]. The score for total burnout is categorised into three categories: high (>42), moderate (34–42), and low (<34) by splitting the score into thirds [20].

#### 2.2.4. Intention to Leave

The Michigan Organisational Assessment Questionnaire (MOAQ) was adopted to assess intention to leave [22]. It has three questions with 5-point Likert scale (strongly disagree to strongly agree; not at all likely to extremely likely). The minimum score was 3, and the maximum score was 15. The reliability of the scale was 0.8 in the previous study and 0.75 in this current study [22]. The intention to leave total score was categorised into three levels: low (M = 1.00–2.33), moderate (M = 2.34–3.66) and high (M = 3.67–5.00). For this, the mean score was divided into thirds due to the absence of categorisation in the original questionnaire [23].

### 2.3. Analyses

Data were analysed using SPSS for Windows version 23.0 [24]. Descriptive statistics using frequencies, means, medians, and standard deviations were obtained for all variables. Normally distributed continuous data are expressed in mean and standard deviation, meanwhile the non-normally distributed data are expressed in median and interquartile ranges. For categorical data, it is expressed in frequency and percentage.

Then, Chi-Square (Fishers Exact), Pearson, or Spearman correlation coefficients were used to evaluate the relationship between researchers’ sociodemographic factors and intention to leave.

For the last research objective, the association between sociodemographic, burnout, and job satisfaction on intention to leave among researchers was studied. A hierarchical multiple regression analysis was applied to determine the most important predictors for intention to leave.

## 3. Results

### 3.1. Sociodemographic Characteristics of the Respondents and Descriptive Results for Research Variables

Table 1 showed the sociodemographic data of the participants and the summary of descriptive statistics of the variables involved. A total of 133 researchers completed the survey. The respondent’s mean age was 38.77 ± 6.35 years old, ranging from 28 to 59 years. The majority of the respondents were females (70.7%), married (74.4%), Malay (78.9%), and had a postgraduate qualification (57.9%). The mean of research working experience was 7.86 ± 5.70 years, and median household income was RM 10,000 ± 7000.

### 3.2. Descriptive Results for Job Satisfaction

**Figure 1:**
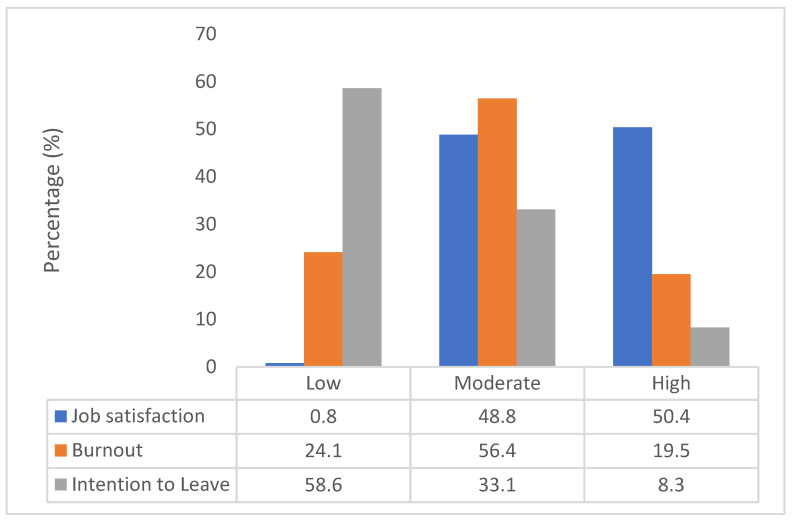
Level of job Satisfaction, burnout, and intention to leave among researchers.

The mean score of the total items was calculated based on a Likert scale of 20 items, ranging from 1 to 5: very dissatisfied (1) to very satisfied (5). The item “The way institute/organisation policies are put into practice” with (M = 3.32, SD = 0.96) reporting the lowest mean score and the highest mean belonging to “The way my job provides for steady employment” with (M = 4.01, SD = 0.74). Job satisfaction total mean was (M = 3.69, SD = 0.62). Most of the respondents were found to be satisfied (99.2%) with their job (Figure 1). The job satisfaction domain is divided into intrinsic, extrinsic, and general (Table 2). The mean for intrinsic was (M = 3.74, SD = 0.63) higher than extrinsic (M = 3.56, SD = 0.75).

### 3.3. Descriptive Results for Burnout

The burnout domain is divided into exhaustion (eight items) and disengagement (eight items) based on a Likert scale, ranging from 1 to 4: strongly agree (1) to strongly disagree (4) of 16 items. The mean of burnout was (M = 2.42, SD = 0.39).

In terms of exhaustion (Table 3), the sub-item “Usually, I can manage the amount of my work well” with (M = 1.86, SD = 0.54) reported the lowest mean score and the highest mean belongs to “The way my job provides for steady employment” with (M = 2.72, SD = 0.98). Meanwhile, for the disengagement, the sub-item “I always find new and interesting aspects in my work (disengagement)” with (M = 1.86, SD = 0.71) and “I find my work to be a positive challenge” with (M = 1.86, SD = 0.66) reported the lowest mean score and the highest mean belongs to “This is the only type of work that I can imagine myself doing” with (M = 2.96, SD = 0.87).

For the high exhaustion component cut off, point score was equal or more than 2.25 and a score of equal or more than 2.10 for high disengagement [21]. For the total burnout score, the threshold values of burnout for the three categories of “low,” moderate,” and “high” were calculated by dividing the score into thirds. The data obtained was divided into 25.50 and 75 percentiles [20]. The returned thresholds for total burnout scores were 34.00 and 42.00, respectively. Thus, the burnout score was classified into low (24.1%), moderate (56.4%), and high (19.5%) (Figure 1). Figure 1 showed that more than two-thirds (75.9%) of the respondents had moderate to high burnout. Table 3 shows the breakdown of the item according to the component of exhaustion and disengagement.

The items for exhaustion and disengagement are ordered from the highest to the lowest mean accordingly. Higher means indicate higher levels of exhaustion or disengagement for each item.

### 3.4. Research Question 1: What Is the Level of Intention to Leave among Researchers

The intention to leave domain of three items was measured based on a Likert scale, ranging from 1 to 5: strongly disagree to strongly agree for the first two items and not at all likely to extremely likely for the last item. As seen in Table 4 with regards to intention to leave, the item “I will probably look for a new job in the next year” with (M = 2.23, SD = 1.24) reported the lowest mean score and the highest mean belongs to “How likely is it that you could find a job with another employer with about the same pay and benefits you now have?” with (M = 2.64, SD = 1.14). The total score for intention to leave item then was categorised into three levels: low (M = 1.00–2.33), moderate (M = 2.34–3.66), and high (M = 3.67–5.00) based on the mean score. Since the absence of categorisation in the original questionnaire, it is divided into thirds [23]. Figure 1 showed more than one-third of the researchers (41.4%) had moderate and high intention to leave the research organisation.

### 3.5. Research Question 2: Do Sociodemographic Factors (Sex, Age, Marital Status, Ethnicity, Educational Level, Research Working Experience, Household Income) Has Any Relationship with the Intention to Leave?

There is no significant association between gender (*p* = 0.077), marital status (*p* = 0.052), and educational status (*p* = 0.360) with the intention to leave (Table 5). Only ethnicity showed a significant association with the intention to leave (*p* = 0.012). Results indicated that the percentage of high intention to leave was higher among non-Malay than Malay.

For numerical data that is not normally distributed, which is household income, the relationship between household income and intention to leave, the Spearman correlation coefficient was used. Meanwhile, other normally distributed variables: age and research working years, were evaluated using Pearson correlation coefficients. Based on Table 6, all three variables have a *p*-value more than 0.05; hence the null hypothesis was accepted. Therefore, it can be concluded that age, household income, and research working years did not linearly correlate with the intention to leave.

### 3.6. Research Question 3: How Would Job Satisfaction, and Burnout Affect the Intention to Leave among Researchers?

For the last research question, the effect of burnout and job satisfaction on intention to leave among researchers was explored. To determine the most important predictors for intention to leave, a hierarchical regression analysis was applied.

The variables with a *p*-value less than 0.25 (gender, marital, and ethnicity) were included in the analysis in the first model based on the *p*-value of bivariate analysis of the sociodemographic. In addition, burnout and job satisfaction were added in the second model.

In model 1, sociodemographic factors (gender, marital status, ethnicity) were entered into the intention to leave model (Table 7). In model 1, F (3,129) = 1.633, *p* > 0.05, indicating the regression model is not significant. All the sociodemographic factors explained 3.7% of the variance in intention to leave (R2 = 0.037). The result showed that gender (β = −0.039, 95% CI (B): 0.427, 0.270), marital (β = −0.135, 95% CI (B): 0.652, 0.077) and ethnicity (β = 0.141, 95% CI (B): −0.070, 0.711) were not significantly related to intention to leave.

In model 2, burnout and job satisfaction were added to the regression model (Table 7). In model 2, F (5,127) = 11.340, *p* < 0.05, indicating the regression model is significant. The R2 = 0.309 revealed that a combination of sociodemographic factors, burnout, and job satisfaction explained 30.9% of the variance in intention to leave. The result showed that gender (β = −0.131, 95% CI (B): −0.568, 0.036), marital (β =−0.093, 95% CI (B): −0.510, 0.117) and ethnicity (β = 0.012, 95% CI (B): −0.318, 0.371) were not significantly related to intention to leave. Meanwhile, the burnout (β = 0.289, 95% CI (B): 0.287, 1.096) and job satisfaction (β = −0.348, 95% CI (B): −0.768, −0.273) were significantly related to the intention to leave).

The results indicate that job satisfaction and burnout are significantly inversely related to intention to leave. Lower job satisfaction is significantly associated with a higher intention to leave among researchers. Meanwhile, higher burnout is significantly related to a higher intention to leave among researchers. Other variables were not significant as the predictors of intention to leave.

There were no collinearity issues among the predictors since VIF was less than the threshold (VIF < 5) and tolerance was more than 0.2. All the result tables are presented below (Table 7).

## 4. Discussion

Although comprehensive research has been carried out on other healthcare professionals, only a very few studies cover medical researchers’ predictors of intention to leave. Thus, this study was carried out to determine the level of intention to leave among the medical researchers and the relationship between sociodemographic characteristics with the intention to leave. The predictors of intention to leave among researchers were also explored.

The findings found a significant effect of job satisfaction and burnout on the intention to leave among researchers. Meanwhile, sociodemographic factors were not significantly associated with the intention to leave.

More than one-third of the researchers had moderate (33.1%) and high (8.3%) intention to leave, which is similar to a study that found more than 40% of healthcare professionals had the intention to leave before the pensionable age [10]. The reasons identified in that similar study were interpersonal relationships, personal factors, and economic factors. Interpersonal relationship is a component of job satisfaction.

This study showed that job satisfaction was negatively associated with the intention to leave. Most of the researchers (99.2%) in this study were satisfied with their job. This is in line with findings of another study that revealed most of the MOH staff (9 out of 10) were satisfied with their jobs [5]. Another study also found that most of the researchers were satisfied with their jobs despite their challenges [25].

Intrinsic factors produced higher job satisfaction compared to extrinsic factors in this study. Intrinsic satisfaction describes how positive individuals feel about their work performance; extrinsic satisfaction relates to extrinsic factors such as financial and recognition or rewards gained through job performance and reputation develop in the working environment [26]. Seven of the highest ranks out of 20 jobs satisfaction items were all intrinsic dimensions of job satisfaction. This includes variety, achievement, social service, activity, independence, ability utilisation, and security, with each item mean scores of 3.77 or higher. This indicated the researchers were more satisfied with the intrinsic factors than extrinsic ones as they felt positive about their job performance. The highest mean (M = 4.01, SD = 0.74) in the job satisfaction domain was the job security item “The way my job provides for steady employment”. This indicated that job security is the most satisfying factor for job satisfaction among researchers, which is similar with a study that found a significant positive relationship between job security and job satisfaction [20]. The possible explanation for this is that government servants are offered stable and permanent employment. Besides that, one of the highest means of job satisfaction items belonged to working conditions (M = 3.87, SD = 0.95). A comfortable and suitable working environment improved job satisfaction among researchers [27].

On the other hand, the way the institute’s policies are put into practice and the way superiors handled their staff reported the lowest mean. This might be due to inappropriate or inadequate policies to accommodate different staff categories. Thus, the management needs to investigate this and reevaluate existing policies. The organisation policies can have a negative impact on the organization [28]. The management needs to tackle the problem as it might affect the researchers’ job satisfaction, hence their decision to leave the institute. In addition to the policies, the role of the supervisor is also important. On top of the policies, the supervisory role is also crucial. Staff who received adequate guidance by their superiors were more likely to be satisfied [29]. Job dissatisfaction could be developed if there is a negative or absence of superior guidance [29], which indirectly could contribute to the researcher leaving the organisation.

The change of the organisation culture is needed to increase the interpersonal relationship between the supervisor or head of department/center and employee. Dysfunctional corporate culture such as low staff morale, bad management style, poor interactions, stress, and a hostile environment should not be tolerated.

Burnout among researchers in the research organisation is also a significant predictor of intention to leave. This is supported by findings that reveal burnout was a predictor of intention to leave [30]. In this study, more than two-thirds (75.9%) of the researchers had moderate and high burnout.

One-third (34.6%) of the researchers experienced high exhaustion, and only 0.8% experienced high disengagement in the component of burnout. This contrasts with findings that revealed the healthcare staff had a higher disengagement to exhaustion during the pandemic COVID-19 [31]. Prolonged exposure to job demands might cause exhaustion [20]. The highest mean was reported for the item “There are days when I feel tired before I arrive at work” (M = 2.72, SD = 0.98). The finding revealed that most of the researchers experienced exhaustion before they arrive at the office for work. This is because they may feel exhausted even before reaching the workplace due to overworking in the previous days or had gone through bad traffic jams.

There are limited studies conducted about burnout among researchers [12]. Many projects conducted among researchers at the time of this study were related to COVID-19. Rapid research output is expected for COVID-19-related projects, which can lead to burnout among the researchers. There are many new areas that need to be explored and there are high demands for the COVID-19 evidence-based research finding. On top of that, there were many limitations and restrictions on research projects during the COVID-19 pandemic [32]. One study found challenges of conducting research during the pandemic as being disrupted by professional networks and working from home [33]. Although some of the researches not related to COVID-19 have been granted extensions to ease deadline pressure, there is often no additional budget involved [33]. Besides, the mobilisation of some of the researchers in the organisation to other healthcare facilities to help the COVID-19 team could cause exhaustion to them and thus result in lesser time to commit to their own prior and existing research task.

Publication is still considered part of the key performance index evaluation in the research organisation even during the pandemic. Sustained high publication pressure and scientific integrity were among the factors identified contributing to burnout among researchers in one of the studies during the non-pandemic COVID-19 era [11]. Burnout was correlated positively with the publication pressure [34]. The emotional exhaustion domain was correlated with burnout symptoms [34]. The scientific output quantity was identified as the cause of the publication pressure, even though this study did not focus on the specific reason for burnout. The effects of Hirsch-index introduction on the scientific field also supported this point [34]. Hence, this indirectly contributed to the cause of burnout among researchers.

In addition, another cause of publication pressure included searching for grants, funding, scholarships, and academic positions [34]. Competitiveness and the increase of unstable careers are among the effects of the publication pressures that can contribute to scientific misconduct and bias [35]. The risk of burnout can also be high if the researchers have a decline in personal well-being. There is a need for the management to address the burnout issues among researchers properly as it could lead to a decline in job performance [34], low-quality research output production or scientific misconduct.

Almost half (49.5%) of those who had moderate and high burnout levels (*n* = 101) had moderate or high intention to leave. Thus, it is crucial for the management to anticipate this problem so that they will not lose the skillful staff and avoid having additional costs related to staff turnover.

In this study, we found that sociodemographic factors (age, gender, marital status, ethnicity, education, and research working years) are not significant predictors of intention to leave. Based on the previous studies on sociodemographic factors, they revealed mixed findings on the association of the sociodemographic factors with the intention to leave. Age is not significantly associated with the intention to leave in this study. This is not in line with a study that found that younger age groups intended to leave the organisation [36]. This study also revealed that gender (β = −0.131, 95% CI (B): −0.568, 0.036) and ethnicity (β = 0.012, 95% CI (B): −0.318, 0.371) were not significantly associated with the intention to leave. This finding is not in line with the studies that found that there was a relationship between gender [37] and ethnicity [38] with the intention to leave. The finding of educational level was in line with a study conducted by Albaqami (2006) that revealed no significant association with intention to leave the organization [39]. Meanwhile, the finding of working years was contradicted with a study by Dachew et al. (2016) in which it was found that working years was significantly associated with intention to leave [40].

Marital status (β = −0.093, 95% CI (B): −0.510, 0.117) also is not significantly associated with intention to leave in this study, which contradicted with a finding of a study that found that singles intended to leave the organisation more so than married people [41]. The relationship between household income and intention to leave also contradicted a study that stated a significant relationship between household income and turnover intention [36].

## 5. Conclusions

One of the limitations of the study is that it is a cross-sectional study, thus it provides only a snapshot of ‘the researcher’s perspective on job satisfaction, burnout, and intention to leave at the point of time when the survey was conducted. The establishment of the causal relationship between all the variables being investigated cannot be done. The possibility of moderacy bias and social desirability bias also might occur as the respondent might answer the option related to the midpoint of the scale. There is also a possibility that some respondents might answer questions that are viewed as good by the interviewer, which lead to underreported or over-reported points about burnout, job satisfaction, or intention to leave.

The questionnaire also covered only certain areas of burnout, job satisfaction, and intention to leave. In addition, response bias might also occur as the questionnaire is a self-reported instrument. If the questions were unclear, no interviewer could explain the questions.

The first challenge encountered for this study was regarding the data collection method. It was changed from a face-to-face to an online survey following the standard operating procedure during the pandemic of Coronavirus Diseases (COVID-19), which required the practice of physical distancing among the respondents. Other than that, there were multiple problems related to the server, which caused some of the respondents to not receive the survey invitation email. Thus, there was a low response rate initially. The problem then was fixed by sending the QR code of the survey link directly to the respondent and inviting the respondent via WhatsApp to maximise the response rate.

Few significant findings can be concluded in this study. It is worrisome to find that more than one-third (41.4%) of the researchers had the intention to leave of moderate to high as the intention to leave is the direct predictor of turnover. Burnout and job satisfaction were the only significant predictors of intention to leave.

A positive relationship was found between burnout and the intention to leave. This means the higher the burnout, the higher the intention to leave among researchers. We found about more than two-thirds (75.9%) of the researchers experienced moderate to high burnout in this organization during COVID-19 pandemic. Most of the researchers experienced a higher exhaustion burnout component compared to disengagement.

Individual and organisational interventions should be implemented to the researchers to reduce burnout among the researchers. Moreover, almost half (49.5%) of those who experienced burnout had moderate to high intention to leave. Thus, it is essential to overcome this problem to prevent the turnover intention, hence reducing turnover-related costs.

Meanwhile, job satisfaction was found to have a negative relationship with the intention to leave. The lower the job satisfaction, the higher intention to leave. The extrinsic factors component was found to have a lower mean compared to the intrinsic factors. The extrinsic factors identified are the organisation’s policy and the interpersonal relationship between the supervisor and the subordinates. The organisation’s policy implementation needs to be reevaluated by the management. Apart from that, the interpersonal relationship between the manager/supervisor with the subordinate needs to be improved. An excellent corporate culture needs to be instilled in the institute to enhance the relationship between both parties. The interpretation of the results of this study must be made cautiously, as the relationships were not adjusted for potential confounding factors.

## Figures and Tables

**Table 1:** Sociodemographic Characteristics of the Respondents and Summary of Descriptive Statistical Results (*n* = 133).

Variables	*n*	(%)	Min	Max	Mean	Median	Standard Deviation	IQR
Age(years) ^a^	133	38.77 (6.35)	28	59	38.77	-	6.35	-
**Gender**								
Male	39	29.3	-	-	-	-	-	-
Female	94	70.7	-	-	-	-	-	-
**Marital status**								
Unmarried (Single/Divorced/Separated/Widowed)	34	25.6	-	-	-	-	-	-
Married	99	74.4	-	-	-	-	-	-
**Ethnicity**								
Malay	105	78.9	-	-	-	-	-	-
Non-Malay	28	21.1	-	-	-	-	-	-
**Highest academic qualification**								
Degree	56	42.1	-	-	-	-	-	-
Post Graduate(Master/PhD)	77	57.9	-	-	-	-	-	-
Research working experience (years) ^a^	133	7.86 (5.70)	1.0	28	7.86	-	5.699	-
Household income(RM) ^b^	124	10,000 (7000)	1000	380	-	10,000	-	7000
Burnout			1.38	3.32	2.42	-	0.389	-
Job Satisfaction			24	100	73.78	-	12.437	-
Intention to Leave			1.00	5.00	2.43	-	0.930	-

Note: a—Mean (SD), b—Median (IQR).

**Table 2:** Descriptive statistics related to job satisfaction (*n* = 133).

	Item	Mean	SD
Intrinsic	The chance to be somebody in the community	3.45	1.00
	The chance to tell people what to do	3.59	0.92
	Being able to do things that don’t go against my conscience	3.64	0.96
	The freedom to use my own judgment	3.65	0.91
	The chance to try my own methods of doing the job	3.65	0.94
	The chance to do different things from time to time	3.77	0.99
	The feeling of accomplishment I get from the job	3.77	0.94
	The chance to do things for other people	3.81	0.91
	Being able to keep busy all the time	3.83	0.81
	The chance to work alone on the job	3.84	0.83
	The chance to do something that makes use of my abilities	3.86	0.92
	The way my job provides for steady employment	4.01	0.74
	Mean for intrinsic	3.74	0.63
Extrinsic	The way institute/organisation policies are put into practice	3.32	0.96
	The way my boss handles his/her workers	3.40	1.03
	The competence of my supervisor in making decision	3.61	1.11
	The praise I get for doing a good job	3.61	0.88
	The chances for advancement on this job	3.66	1.07
	My pay and the amount of work I do	3.74	1.01
	Mean for extrinsic	3.56	0.75
General	The way my co-workers get along with each other	3.71	0.93
	The working conditions	3.87	0.95
	Total	3.69	0.62

The intrinsic and extrinsic job satisfaction items are ordered from the lowest to the highest mean accordingly. Lower means indicate lower levels of job satisfaction for each item.

**Table 3:** Descriptive statistics related to burnout (*n* = 133).

	Item	Mean	SD
Exhaustion	There are days when I feel tired before I arrive at work	2.72	0.98
	After my work, I usually feel worn out and weary	2.68	0.82
	After work, I tend to need more time than in the past to relax and feel better	2.51	0.97
	After working, I have enough energy for my leisure activities	2.39	0.84
	During my work, I often feel emotionally drained	2.2	0.87
	When I work, I usually feel energised	2.11	0.68
	I can tolerate the pressure of my work very well	1.95	0.65
	Usually, I can manage the amount of my work well	1.86	0.54
	Mean for Exhaustion	2.71	0.28
Disengagement	This is the only type of work that I can imagine myself doing	2.96	0.87
	Sometimes I feel sickened by my work tasks	2.28	0.95
	Over time, one can become disconnected from this type of work	2.19	0.95
	Lately, I tend to think less at work and do my job almost mechanically	2.14	0.73
	It happens more and more often that I talk about my work in a negative way	2.08	0.86
	I feel more and more engaged in my work	1.98	0.74
	I always find new and interesting aspects in my work	1.86	0.71
	I find my work to be a positive challenge	1.86	0.66
	Mean for Disengagement	2.25	0.47
	Total	2.42	0.39

**Table 4:** Descriptive statistics for intention to leave (*n* = 133).

Item	Mean	SD
I will probably look for a new job in the next year	2.23	1.24
I often think about quitting	2.43	1.31
How likely is it that you could find a job with another employer with about the same pay and benefits you now have?	2.64	1.14
Total	2.43	0.93

**Table 5:** Association between marital status, educational level, ethnicity, gender and intention to leave.

Variable	Low *n* (%)	Moderate *n* (%)	High *n* (%)	*p*-Value
**Gender**				0.077
Male	24 (61.5)	9 (23.1)	6 (15.4)	
Female	54 (57.4)	35 (37.2)	5 (5.3)	
**Marital status**				
Not Married	15 (44.1)	17 (50.0)	2 (5.9)	0.052
Married	63 (63.6)	27 (27.3)	9 (9.1)	
**Educational level**				0.360
Degree	29 (51.8)	21 (37.5)	6 (10.7)	
Master/PhD	49 (63.6)	23 (29.9)	5 (6.5)	
**Ethnicity**				0.012
Malay	62 (59.0)	38 (36.2)	5 (4.8)	
Non-Malay	16 (57.1)	6 (21.4)	6 (21.4)	

**Table 6:** The correlation between age, research working experience, and household income with the intention to leave.

Variable	Intention to Leave	
	r	*p*-Value
Age ^a^	0.029	0.738
Research working experience ^a^	−0.095	0.274
Household income ^b^	0.033	0.717

^a^ Pearson correlation coefficient, ^b^ Spearman correlation coefficient.

**Table 7:** Results of hierarchical regression analysis for intention to leave.

Variables	Model 1					Model 2					Tolerance	VIF
	B (95% CI)	SE	Beta	t	*p* Value	B (95% CI)	SE	Beta	t	*p*-Value		
(Constant)	2.681	0.497		5.395	<0.001	3.446	0.917		3.758	0.001		
Gender	−0.078 (−0.427, 0.270)	0.176	−0.039	−0.446	0.657	−0.266 (−0.568, 0.036)	0.153	−0.131	−1.740	0.084	0.967	1.034
Marital status	−0.288 (−0.652, 0.077)	0.184	−0.135	−1.560	0.121	−0.197 (−0.510, 0.117)	0.159	−0.093	−1.239	0.218	0.977	1.024
Ethnicity	0.321 (−0.070, 0.711)	0.197	0.141	1.624	0.107	0.027 (−0.318, 0.371)	0.174	0.012	0.154	0.878	0.929	1.076
Job satisfaction						−0.520 (−0.768, −0.273)	0.125	−0.348	−4.160 *	<0.001	0.778	1.285
Burnout						0.692 (0.287, 1.096)	0.205	0.289	3.382 *	0.001	0.745	1.342
F value	1.633					11.340						
*p* value	0.185					<0.001						
R2 (Adj R2)	0.037 (0.014)					0.309 (0.281)						

* *p* < 0.005.

## Data Availability

Not applicable.
