# Correction: Mat Rifin, M.; Danaee, M. Association between Burnout, Job Dissatisfaction and Intention to Leave among Medical Researchers in a Research Organisation in Malaysia during the COVID-19 Pandemic. *Int. J. Environ. Res. Public Health* 2022, *19*, 10017

**DOI:** 10.3390/ijerph20031882

**Published:** 2023-01-19

**Authors:** Halizah Mat Rifin, Mahmoud Danaee

**Affiliations:** 1 Institute for Public Health, National Institutes of Health, Ministry of Health Malaysia, Shah Alam 40170, Malaysia; 2 Department of Social and Preventive Medicine, Faculty of Medicine, University Malaya, Kuala Lumpur 50603, Malaysia; mdanaee@um.edu.my

## Error in Table

In the original publication [1], the value ‘10,759.77’ in the standard deviation of the Research working experience (years) variable was wrong and should have been ‘5.699’. The revised version of Table 1 is as below. The authors state that the scientific conclusions are unaffected. This correction was approved by the Academic Editor.

In the original publication, there was an error in Table 5 as published in the main manuscript under Section 3 Results, Section 3.5; the p-value of 0.083 for the Gender variable was incorrect and should have been 0.077; the p-value of 0.055 for the Marital status variable was incorrect and should have been 0.052; the p-value of 0.385 for the educational level variable was incorrect and should have been 0.360; the value of 16.7% under the High Intention to Leave column among those of the male gender was incorrect and should have been 15.4%; the value of 5,3 under High Intention to Leave column among those of the female gender was incorrect and should have been 5.3%; the value 279 under Moderate Intention to Leave column among those married row was incorrect and should have been 27, the value of 44 (33.1) under Moderate Intention to Leave column of the Non-Malay row should have been 6 (21.4); and the value of 11 (8.3) under High Intention to Leave column of the non-Malay row was incorrect and should have been 6 (21.4). The revised version of Table 5 is below. The authors state that the scientific conclusions are unaffected. This correction was approved by the Academic Editor. The original publication has also been updated.

In the original publication, there was an error in Table 7 as published in the main manuscript under Section 3 Results, Section 3.6: the lower bound of 95% CI value 0.427 for the Gender variable was incorrect and should have been −0.427 and the lower bound of 95% CI value 0.652 for the Marital status was incorrect and should have been −0.652.

## Text Correction

There was an error in the original publication under Section 2. Materials and Methods, Section 2.1. Participants, paragraph 4 and paragraph 5.

In paragraph 4, the statement “The sample population was 149 based on the sample size recommendation from G* Power Software (114 + 30% non-response rate) out of 588 researchers in the organisation. An additional 30% of the sample size was added to secure the final sample size in case of refusal” was incorrect.

A correction has been made to Section 2, Section 2.1, paragraph number 4.

“The sample population was 149 based on the sample size recommendation from G* Power Software (118 + 25% non-response rate) out of 588 researchers in the organisation. An additional 25% of the sample size was added to secure the final sample size in case of refusal”.

In paragraph 5, the statement “Of those, 149 researchers were selected after considering 30% of the non-response rate.” was incorrect.

A correction has been made to Section 2, Section 2.1, paragraph number 5.

“Of those, 149 researchers were selected after considering 25% of the non-response rate.”

There was an error in the original publication in the statement as published under Section 3.5, paragraph 1. The statement “There is no significant association between gender (*p* = 0.083), marital status(*p* = 0.055), and educational status (*p* = 0.385) with the intention to leave (Table 5)” was incorrect.

A correction has been made to Section 3, Section 3.5, paragraph number 1:

“There is no significant association between gender (*p* = 0.077), marital status (*p* = 0.052), and educational status (*p* = 0.360) with the intention to leave (Table 5)”.

There was an error in the original publication in the statement as published under Section 3.6, paragraph 3. The statement “The result showed that gender (β = −0.039, 95% CI (B): −0.427, 0.270), marital (β = −0.135, 95% CI (B): −0.652, 0.077) and ethnicity(β = 0.141, 95% CI (B): −0.070, 0.711) were not significantly related to intention to leave” was incorrect.

A correction has been made to Section 3, Section 3.6, paragraph 3:

“The result showed that gender (β = −0.039, 95% CI (B): 0.427, 0.270), marital (β = −0.135, 95% CI (B): 0.652, 0.077) and ethnicity (β = 0.141, 95% CI (B): −0.070, 0.711) were not significantly related to intention to leave”.

There was also an error in the original publication in the statement as published under Section 3.6, paragraph 4. The statement “Meanwhile, the burnout (β = 0.285, 95% CI (B): 0.272, 1.093) and job satisfaction (β = −0.348, 95% CI (B): −0.768, −0.273) were significantly related to the intention to leave)” was incorrect.

A correction has been made to Section 5, Section 3.6, paragraph number 4:

“Meanwhile, the burnout (β = 0.289, 95% CI (B): 0.287, 1.096) and job satisfaction (β = −0.348, 95% CI (B): −0.768, −0.273) were significantly related to the intention to leave)”.

There was also an error in the original publication in the statement as published under Section 4, paragraph 7. The statement “The change of the organisation culture is needed to increase the interpersonal relationship between the supervisor or head of department/center” was incorrect.

A correction has been made to Section 4, paragraph 7:

“The change of the organisation culture is needed to increase the interpersonal relationship between the supervisor or head of department/center and employee”.

The authors state that the scientific conclusions are unaffected. This correction was approved by the Academic Editor. The original publication has also been updated.

## Figures and Tables

**Table 1:** Sociodemographic Characteristics of the Respondents and Summary of Descriptive Statistical Results (*n* = 133).

Variables	*n*	(%)	Min	Max	Mean	Median	Standard Deviation	IQR
Age(years) ^a^	133	38.77 (6.35)	28	59	38.77	-	6.35	-
**Gender**								
Male	39	29.3	-	-	-	-	-	-
Female	94	70.7	-	-	-	-	-	-
**Marital status**								
Unmarried (Single/Divorced/Separated/Widowed)	34	25.6	-	-	-	-	-	-
Married	99	74.4	-	-	-	-	-	-
**Ethnicity**								
Malay	105	78.9	-	-	-	-	-	-
Non-Malay	28	21.1	-	-	-	-	-	-
**Highest academic qualification**								
Degree	56	42.1	-	-	-	-	-	-
Post Graduate(Master/PhD)	77	57.9	-	-	-	-	-	-
Research working experience (years) ^a^	133	7.86 (5.70)	1.0	28	7.86	-	5.699	-
Household income(RM) ^b^	124	10,000 (7000)	1000	380	-	10,000	-	7000
Burnout			1.38	3.32	2.42	-	0.389	-
Job Satisfaction			24	100	73.78	-	12.437	-
Intention to Leave			1.00	5.00	2.43	-	0.930	-

Note: a—Mean (SD), b—Median (IQR).

**Table 5:** Association between Marital Status, Educational Level, Ethnicity, Gender and Intention to Leave.

Variable	Low *n* (%)	Moderate *n* (%)	High *n* (%)	*p*-Value
**Gender**				0.077
Male	24 (61.5)	9 (23.1)	6 (15.4)	
Female	54 (57.4)	35 (37.2)	5 (5.3)	
**Marital status**				
Not Married	15 (44.1)	17 (50.0)	2 (5.9)	0.052
Married	63 (63.6)	27 (27.3)	9 (9.1)	
**Educational level**				0.360
Degree	29 (51.8)	21 (37.5)	6 (10.7)	
Master/PhD	49 (63.6)	23 (29.9)	5 (6.5)	
**Ethnicity**				0.012
Malay	62 (59.0)	38 (36.2)	5 (4.8)	
Non-Malay	16 (57.1)	6 (21.4)	6 (21.4)	

**Table 7:** Results of Hierarchical Regression Analysis for Intention to Leave.

Variables	Model 1					Model 2					Tolerance	VIF
	B (95% CI)	SE	Beta	t	*p* Value	B (95% CI)	SE	Beta	t	*p*-Value		
(Constant)	2.681	0.497		5.395	<0.001	3.446	0.917		3.758	0.001		
Gender	−0.078 (−0.427, 0.270)	0.176	−0.039	−0.446	0.657	−0.266 (−0.568, 0.036)	0.153	−0.131	−1.740	0.084	0.967	1.034
Marital status	−0.288 (−0.652, 0.077)	0.184	−0.135	−1.560	0.121	−0.197 (−0.510, 0.117)	0.159	−0.093	−1.239	0.218	0.977	1.024
Ethnicity	0.321 (−0.070, 0.711)	0.197	0.141	1.624	0.107	0.027 (−0.318, 0.371)	0.174	0.012	0.154	0.878	0.929	1.076
Job satisfaction						−0.520(−0.768, −0.273)	0.125	−0.348	−4.160 *	<0.001	0.778	1.285
Burnout						0.692 (0.287, 1.096)	0.205	0.289	3.382 *	0.001	0.745	1.342
F value	1.633					11.340						
*p* value	0.185					<0.001						
R2 (Adj R2)	0.037 (0.014)					0.309 (0.281)						

* *p* < 0.005.
